# Assessment of antibiotic prescribing quality through repeated point prevalence surveys in a Malaysian teaching hospital

**DOI:** 10.1017/ash.2025.154

**Published:** 2025-09-03

**Authors:** Nurul Adilla, Hayat Jamaluddin, Isa Naina Mohamed, Petrick Periyasamy, Lau Chee Lan, Najma Kori, Ramliza Ramli, Tan Toh Leong, Yin Mei Kuen, Nur Jannah Azman, Sasheela Ponnampalavanar, Rodney James, Karin Thursky

**Affiliations:** 1 Pharmacoepidemiology and Drug Safety Unit, Department of Pharmacology, Faculty of MedicineUniversiti Kebangsaan Malaysia, 56000 Cheras, Kuala Lumpur, Malaysia; 2 Department of Hospital and Clinical Pharmacy, Faculty of Pharmacy University of Cyberjaya, Cyber 11, 63000 Cyberjaya, Selangor, Malaysia, Medical Department Universiti Kebangsaan Malaysia Medical Centre (UKMMC), Cheras, Kuala Lumpur, Malaysia; 3 Medical Department, Faculty of MedicineUniversiti Kebangsaan Malaysia, 56000 Cheras, Kuala Lumpur, Malaysia; 4 Pharmacy DepartmentHospital Canselor Tuanku Muhriz, 56000 Cheras, Kuala Lumpur, Malaysia; 5 Department of Medical Microbiology and Immunology, Faculty of MedicineUniversiti Kebangsaan Malaysia, 56000 Cheras, Kuala Lumpur, Malaysia; 6 Department of Emergency Medicine, Faculty of MedicineUniversiti Kebangsaan Malaysia, 56000 Cheras, Kuala Lumpur, Malaysia; 7 Department of MedicineUniversity of Malaya, 50603 Kuala Lumpur, Malaysia; 8 National Centre for Antimicrobial StewardshipPeter Doherty Research Institute for Infection and Immunity, 3000 Melbourne, Australia

## Abstract

**Introduction:** Optimising antibiotic prescribing in hospitals through antimicrobial stewardship (AMS) initiatives is essential in addressing the global threat of antimicrobial resistance. **Methods:** Point prevalence surveys were performed in April 2019 and November 2022 utilizing the Hospital National Antimicrobial Prescribing Survey (NAPS) tool. The study aimed to evaluate the prevalence of antibiotic use among inpatients and monitor antibiotic prescribing quality in 2022 compared to 2019 in a Malaysian teaching hospital as part of AMS core elements. **Results:** The prevalence of antibiotic use remained relatively stable between 2019 and 2022 (44.1% vs. 42.3%), with no significant change observed. Prescription patterns, including the type of antimicrobials, treatment modalities, and prescriptions per patient, showed insignificant differences between the two surveys. Antibiotics from the World Health Organisation (WHO) Access group constituted up to 47% of prescriptions in 2022, while usage of antibiotics from Watch group decreased from 57% to 53%, albeit insignificantly. Notably, there was a non- significant increase in appropriate prescribing for surgical prophylaxis in 2022 (40% vs 16.7%, p=0.078), alongside a less prevalent in prolonged surgical prophylaxis (28% in 2022 vs. 50% in 2019). Despite static prevalence and prescribing patterns, compliance with guidelines (p<0.006) and appropriate prescribing (p<0.002) showed significant improvement. The likelihood of compliance and appropriate prescribing was approximately 1.8-fold higher in 2022 compared to 2019. However, an increase in prescription of unnecessary broad-spectrum antibiotics was observed (23.1% vs 48%, p=0.002). Multiple logistic regression revealed that inappropriate prescribing significantly occurred when antibiotic indication was poorly documented (adjusted OR 3.67;95% CI 1.28–10.53; p=0.016). **Conclusion** : While prescribing patterns remained relatively unchanged, our findings highlight notable improvement in antibiotic prescribing quality. However, challenges persist, including the increased use of unnecessary broad-spectrum antibiotics. Continued efforts in AMS are imperative to address these issues and further enhance prescribing practices.

